# 人工神经网络在肺癌相关研究中的应用现状

**DOI:** 10.3779/j.issn.1009-3419.2019.04.08

**Published:** 2019-04-20

**Authors:** 星宇 朱, 楠 陈

**Affiliations:** 1 610041 成都，四川大学华西临床医学院 West China School of Medicine, Sichuan University, Chengdu 610041, China; 2 610041 成都，四川大学华西医院胸外科 Department of Toracic Surgery, West China Hospital, Sichuan University, Chengdu 610041, China

**Keywords:** 肺癌, 精准医学, 人工神经网络, Lung neoplasms, Precision medicine, Artifcial neural networks

## Abstract

肺癌是目前全世界发病率、死亡率最高的肿瘤，目前的诊疗手段效果有限，精准医学的全面开展为提高肺癌诊疗水平带来了新的契机。但临床医生很难对精准医学需要的多维度多角度的资料（生物组学、临床检测指标以及非生物的环境背景资料等）进行有效的整合和利用，难以为患者选择最优的诊治方案。借助计算机技术的发展，以人工神经网络（artificial neural networks, ANNs）为代表的人工智能具有高容错性、智能性和具有自我学习能力的特点，其强大的信息整合能力可以对精准医学的发展与应用起到很大的帮助，在肺癌的基础研究和临床实践中发挥巨大的作用。本文对肺癌领域ANNs应用的现状进行综述。

## Abstract (en)

Lung cancer is the most common and fatal tumor in the world with limited diagnostic and treatment methods. Te development of precision medicine has brought new opportunities for the improvement of diagnosis and treatment of lung cancer. However, various kinds of information required by precision medicine (such as biometrics, clinical test indicators and non-biological environmental background information) are difcult for clinicians to integrate and utilize e?ectively. With the development of computer technology, artifcial neural networks (ANNs), which has the characteristic of high fault tolerance, intelligence and self-learning ability. Its powerful information integration ability can solve many problems in the development and application of precision medicine, which can play a key role in basic research and clinical practice associated with lung cancer. Tis article reviewed the application of artifcial neural network in the feld of lung cancer.

世界卫生组织国际癌症研究署（International Agency for Research on Cancer, IARC）发布的GLOBOCAN2018癌症报告显示^[1]^肺癌是全球发病率最高及死亡人数最多的恶性肿瘤，虽然在所有癌症中肺癌诊断率最高，但大多数患者已处于终末期。随着精准医学的兴起，提高肺癌早期诊断率及实现个体化的诊疗有了新的可能。精准医学通过整合患者的各项资料，经过大数据分析，进行精准诊断，找到最适合患者的治疗靶点及方案，并有效评估预后，最终实现提高肺癌疗效、改善患者生活质量的目标^[2]^。但精准医学需要收集患者各种生物组学、临床检测指标以及其他不同的环境背景资料，存在数据量过于庞大且彼此独立难以建立合适的数学模型进行有效的统计分析等问题，如何将大数据应用于临床和科研是目前的一大难题，也是研究的重点方向之一。人工神经网络（artificial neural networks, ANNs）是现代计算机人工智能（artificial intelligence, AI）最重要的分支，其最大的能力在于整合已有的海量信息，提高人们分析、处理信息的效率。通过机器学习对相关资料进行大数据的整合与分析，将有助于解决目前肺癌精准医学发展所遇到问题。为此，本文对肺癌领域ANNs应用的现状进行综述。

## 1 人工神经网络现状

### 1.1 人工神经网络兴起发展历程

1943年，美国心理学家McCulloch与数学家Pitts合作^[3]^，用逻辑数学工具研究神经网络的过程中，首次提出了神经元的数学模型，简称为MP模型，从此开启了对神经网络的理论研究。经过以感知器（Perceptrons）为代表的第一代ANNs^[4, 5]^，和以Hopfield网络和BP网络为代表的第二代ANNs^[6, 7]^的发展，目前的第三代ANNs以机器深度学习（deep machine learning, DML）为特点^[8]^。DML的研究关注的是与大脑皮层信息表达相似的计算模型，有卷积神经网络（convolutional neural networks, CNNs）和深度置信网（deep belief nets, DBNs）两种主流的方法，这两种方法区别在于CNNs是一种有监督学习的机器学习模型，而DBNs是一种无监督学习的机器学习模型^[9]^。

### 1.2 人工神经网络概念、特点

ANNs是一种模拟大脑神经元细胞传递信息构建的模型，在对人脑结构及其对外界刺激的响应机制进行理解和抽象后，以网络拓扑为理论基础将数据进行非线性建模，从而模拟人脑对复杂信息的处理模式，具有高容错性、智能性、能够自我学习等特征^[10]^。与数字计算机相比，ANNs在构成原理和功能特点等方面更加接近人脑，它并不按既定的程序逐步执行运算，而是能够通过自我学习，总结规律，从而去完成运算、识别或过程控制等任务。DML是目前ANNs最大的特点，是一种特征学习方法，能够把原始数据通过一些简单的但是非线性的模型转变成为更高层次的、更加抽象的表达，通过足够多转换的组合，非常复杂的函数也可以被机器学习。

### 1.3 人工神经网络在医学领域的运用

ANNs在医学领域的应用十分广泛，20世纪90年代开始ANNs在诊断、影像分析、心电图分析、预后评估、对药物疗效的反应等方面均有相应的研究和运用^[11]^。近年来也有ANNs运用于计算机临床决策支持系统（Clinical Decision Support System, CDSS）的报道^[12]^。在皮肤癌诊断、内镜图像诊断、精神病学研究、视网膜OCT图像诊断中，ANNs的应用取得了不错的进展^[13-16]^，诊断方面在部分领域甚至能达到有经验的临床专家的水平。此外ANNs在虚拟助理、药物发掘、营养学、生物技术、急救室/医院管理、健康管理、精神健康、卫生经济学、可穿戴设备开发、风险管理和病理学等领域有着不同程度的运用。

## 2 人工神经网络在肺癌相关研究中的应用

### 2.1 肺癌诊断与分期

由于肺癌早期没有特异的临床症状且肿瘤常位于深部被正常组织包裹，使得肺癌的早期诊断是肺癌诊疗中最具有挑战性的工作。基于ANNs模式识别有非常高的肺癌辅助诊断价值^[17]^。目前研究大多将影像学图像、基因表达谱、临床资料或组织病理学测定等资料纳入作为ANNs的输入变量，通过对变量进行选择和组合，或构建不同的神经网络及算法，使诊断的准确度得到提升^[18]^。

#### 2.1.1 影像学

影像学是目前应用最广泛的肺癌筛查与早期诊断技术。随着影像技术的发展图像的清晰度及分辨率逐步提升，图像中蕴涵的信息变得越来越丰富，需要有经验的影像医生花费大量的时间与精力去详细解读，但基于人眼的识别存在遗漏关键的信息的风险，导致误诊及漏诊^[19]^。ANNs有强大的图像分析能力，可以快速识别关键信息进行分析，并减少信息遗漏。Shen等^[20]^运用多尺度卷积神经网络（multiple convolutional neural network, MCNN）分析胸部CT图像上的结节，经过704份良性结节和396份恶性结节图像的训练后，用另外275份图像进行测试，对结节的良恶性的判断准确率达86.84%。Liu等^[21]^构建的CT图像计算机辅助诊断系统，能识别图像中89.4%的孤立结节、胸膜旁结节、血管旁结节、磨玻璃样结节（ground glass opacity, GGO），检出其他结节（如炎性结节）带来的假阳性在每例病案中能控制在2个结节以下。Toney等^[22]^利用133例非小细胞肺癌患者的PET-CT图像，通过ANNs和影像专家分别对淋巴结转移进行分期（N0、N1、N2、N3），再与术后病理分期作为金标准进行比较，结果显示淋巴结分期ANNs的准确率高达99.2%，而影像专家为72.4%。目前对于ANNs的分析结果仍需影像医师的审阅，随着ANNs算法的优化和利用大数据进行训练，ANNs利用影像进行诊断的准确度将逐步提高，甚至做到在临床环境中达到乃至超过有经验的影像医师的正确率，使影像结果判读做到高水准的标准化、同质化。在提高诊断正确率的同时还能使不同医院间检查结果达到互信，避免患者重复接受检查，具有卫生经济学的现实意义。

#### 2.1.2 生物标志物

Duan等^[23]^纳入200例不同病理类型的各期别原发性肺癌患者和200名正常对照，分别收集患者性别、年龄、吸烟史等临床资料以及*p16*、*RASSF1A*、*FHIT*启动子三种基因的甲基化水平和相对端粒长度，将其中148例患者和152名正常对照的资料用于ANNs学习训练，将其余受试者资料进行测试，结果显示诊断准确率相较于采用线性分析模型有所提高（76% *vs* 67%）。该研究提示运用ANNs能通过分析多种基因提高辅助诊断的准确率。Butcher等^[24]^运用ANNs构建的多层感知机（multi-layer perceptron, MLP），对使用选择离子流动管质谱仪（SIFT-MS）测得的20例肺癌患者和20名健康受试者呼出气体挥发性有机化合物（volatile organic compounds, VOCs）浓度进行分析，将15种VOCs用于肺癌诊断准确率可达74%。Tomasz等^[25]^运用ANNs分析固相微萃取-气相色谱-质谱联用（solid phase microextraction-gas chromatography-mass spectrometry, SPME-GC/MS）技术检测到的VOCs，选出8种VOCs用于肺癌诊断的敏感度为63.5%，特异度为72.4%。但这两项关于VOCs的研究样本量有限且并未对长期吸烟等高危因素进行亚组分析，可将临床资料纳入作为ANNs的变量或许准确度能得到进一步的提升。此外也有运用ANNs分析痰液中非小细胞肺癌生物标志物用于辅助诊断的报告^[26]^。随着检验技术的提升，特别是生物芯片等技术的出现，将有大量例如DNA片段、抗原抗体、microRNA等生物标志物被检出，而ANNs能对分析这些标志物与疾病之间的关系起到非常大的帮助。

#### 2.1.3 病理及危险因素分析

Alzubaidi等^[27]^总结了7项基于ANNs的数字病理学在肺癌领域中的研究情况，多项研究利用组织学或细胞学特征对肺癌进行诊断，准确率可达50%-98%。当术中根据手术视频进行快速分析时，大流量的数据会超过部分研究所用ANNs的分析限度，存在一定的局限性。对于切片，高的诊断准确率依赖于高质量的切片图像，且对于连续多张切片需要尽量减少非期望部位的采样，以提高诊断准确率。因此，建立可靠的分析模型，提高显微镜下取得的图像质量增加以及开发可满足巨大样本量存储和计算分析的设备是提升ANNs诊断准确率的有效途径。在肺癌易感性和相关危险因素分析方面，Xie等^[28]^应用ANNs分析不同危险因素与肺癌发生的具体关系，对41项危险因素进行分析后发现，在纳入其中15项危险因素作为预测指标时，预测肺癌发生的准确率可达83.816%。通过ANNs分析整合与肺癌确切相关的危险因素，可筛选出发病的高危人群，并对这部分人群的危险因素进行早期干预是降低肺癌发病率的有效且具有应用前景的一种方法。

### 2.2 肺癌预后和治疗判断

过去20年有多种不同的ANNs模型应用于肺癌患者的疗效预测和预后判断，目标是能够了解不同的干预措施能对患者带来的获益，对癌症复发率及生存率进行预判^[18]^，为患者选择最优的个体化治疗方案。

#### 2.2.1 预后预测

Hsia等^[29]^将临床检测指标与基因多态性检测结果联合纳入，通过ANNs构建模型预测75例无手术治疗指征的肺癌患者预后并依此制定治疗方案。患者实际平均生存期为（12.44±7.95）个月，而ANNs预测结果为（13.16±1.77）个月，准确率可达86.2%。Paul等^[30]^应用ANNs分析肺癌患者CT图像的特征后对患者预后进行预测，选取十种不同的形态特征时，准确率为77.5%，进一步选取特异性最高的五种形态特征与数量特征结合，预测准确率提高到82.5%。Chatzimichail等^[31]^应用ANNs对经过手术治疗后的非小细胞肺癌患者进行预后预测时发现，当把γ-H2AX表达情况加入后能提高预测的准确率，提示γ-H2AX可作为评估早期非小细胞肺癌预后的生物标志物。该研究表明可通过验证ANNs预测预后准确度的不同判断预后预测指标的有效性。通过ANNs分析预测个体的生存时间，可指导医生和患者共同选择和制定合理的治疗方案，从而减少过度治疗和不必要的侵入性医疗操作，并可提高患者生存质量以及指导卫生经济学决策。

#### 2.2.2 并发症预测

肺癌患者术后出现并发症的几率并不一致，如果能早期判断并发症的可能性，并据此对患者进行危险性分层，可对高危患者进行有针对性的干预，并减少对低危患者的过度治疗。Santos-García等^[32]^应用设计的ANNs模型预测489例非小细胞肺癌患者肺叶或肺切除术后呼吸循环系统并发症的发生情况，准确率达98%。Chen等^[33]^应用ANNs分析肺癌患者年龄、抗生素使用情况、血清白蛋白浓度、是否接受化疗或手术、血红蛋白浓度以及住院时间等因素，预测患者是否会发生深部真菌感染，准确率达82.9%。

#### 2.2.3 治疗方案确定

辅助化疗对于预防非小细胞肺癌患者术后复发或转移的效果仍存在争议，Chen等^[34]^利用ANNs结合T分期和相关基因表达情况进行分析，预测辅助化疗是否对预后的有改善，发现DUSP6和LCK的表达对预后预测的准确率为65.71%。该研究表明利用ANNs分析某些基因的表达情况可指导辅助化疗的使用，避免无效的干预和减少医疗资源的浪费，避免增加患者药物带来的副反应，未来可利用ANNs选择更好的基因位点进行分析，以提高预测的准确率。目前智能决策中比较成熟的沃森肿瘤系统（Watson for oncology, WFO），是由IBM基于ANNs开发的认知计算系统，有国内的研究表明该系统对于肺癌的临床决策与中国专家团队有较高的一致性^[35]^。WFO只是辅助医疗工具，在一些实际应用中却被用于对病人做出临床的直接诊断，而未经医生详细的评估审核，在智能还没有完全可以取代医生之前，这样的尝试会增加患者的疑虑，且WFO提供的诊疗方案仅是方向性的框架，仍然需要医生去细化执行。此外，目前WFO缺乏中国本土化的真实病例、文献等进行训练，是否适用于中国的医疗流程与环境还待进一步的检验。

## 3 总结与展望

通过收集整合患者的各种生物组学、临床检测指标以及非生物的环境背景资料建立大数据资料库是信息时代医学研究的重点方向之一。对这些数据进行有效的分析和解读将是重中之重，而对已有的海量信息的整合、分析恰恰是ANNs最大的优势。目前国内外对ANNs在肺癌及整个医学领域的投入巨大，但离真正的临床应用还有一定的距离。缺少高质量的标准化肺癌临床数据库是制约ANNs用于肺癌领域的研究的重要因素，不仅影响准确性，而且是不同系统能够相比较的关键点。目前的研究样本量普遍有限，绝大多数预测或诊断研究只在两种或少数几种疾病中评估的准确度，脱离临床实际环境，限制了临床应用的价值。有研究指出目前医学领域运用ANNs缺乏正确的方法和评判标准，结果的可信度存疑^[36]^。在社会、法规方面，诊疗结果医疗责任问题、信息安全问题等还缺乏通行的技术规范。未来可由各大中心牵头建立多中心的标准化肺癌临床数据库，作为符合中国流行病学的国家级信息库，并以此开发符合中国临床环境的ANNs系统，这对提高基层医院及体检中心肺癌检出率，完善三级诊疗以及优化医疗资源都有积极意义。另一方面，积极推行针对ANNs的制度法规、完善技术规范及审核制度，为ANNs的发展提供制度的支持和相应的制约。未来ANNs用于肺癌研究具有美好前景，但仍充满挑战。
